# Differentiation between depression and bipolar disorder in child and adolescents by voice features

**DOI:** 10.1186/s13034-024-00708-0

**Published:** 2024-01-29

**Authors:** Jie Luo, Yuanzhen Wu, Mengqi Liu, Zhaojun Li, Zhuo Wang, Yi Zheng, Lihui Feng, Jihua Lu, Fan He

**Affiliations:** 1 National Clinical Research Center for Mental Disorders, Beijing Key Laboratory of Mental Disorders, Beijing Anding Hospital, Beijing Institute for Brain Disorders Capital Medical University, De Sheng Men Wai An Kang Hu Tong 5 Hao, Xi Cheng Qu, Beijing, 100088 People’s Republic of China; 2 Beijing Institute of Technology, School of Integrated Circuits and Electronics, Zhongguancun South Street 5 Hao, Hai Dian Qu, Beijing, 100081 China; 3 Beijing Institute of Technology, School of Optics and Photonics, Zhongguancun South Street 5 Hao, Hai Dian Qu, Beijing, 100081 China

**Keywords:** Mood disorder, Voice features, Diagnosis, Child and adolescent, Classification accuracy

## Abstract

**Objective:**

Major depressive disorder (MDD) and bipolar disorder (BD) are serious chronic disabling mental and emotional disorders, with symptoms that often manifest atypically in children and adolescents, making diagnosis difficult without objective physiological indicators. Therefore, we aimed to objectively identify MDD and BD in children and adolescents by exploring their voiceprint features.

**Methods:**

This study included a total of 150 participants, with 50 MDD patients, 50 BD patients, and 50 healthy controls aged between 6 and 16 years. After collecting voiceprint data, chi-square test was used to screen and extract voiceprint features specific to emotional disorders in children and adolescents. Then, selected characteristic voiceprint features were used to establish training and testing datasets with the ratio of 7:3. The performances of various machine learning and deep learning algorithms were compared using the training dataset, and the optimal algorithm was selected to classify the testing dataset and calculate the sensitivity, specificity, accuracy, and ROC curve.

**Results:**

The three groups showed differences in clustering centers for various voice features such as root mean square energy, power spectral slope, low-frequency percentile energy level, high-frequency spectral slope, spectral harmonic gain, and audio signal energy level. The model of linear SVM showed the best performance in the training dataset, achieving a total accuracy of 95.6% in classifying the three groups in the testing dataset, with sensitivity of 93.3% for MDD, 100% for BD, specificity of 93.3%, AUC of 1 for BD, and AUC of 0.967 for MDD.

**Conclusion:**

By exploring the characteristics of voice features in children and adolescents, machine learning can effectively differentiate between MDD and BD in a population, and voice features hold promise as an objective physiological indicator for the auxiliary diagnosis of mood disorder in clinical practice.

**Supplementary Information:**

The online version contains supplementary material available at 10.1186/s13034-024-00708-0.

## Introduction

Major Depressive Disorder (MDD) and Bipolar Disorder (BD) are two common mood disorders that severely affect the physical and mental health of children and adolescents [1]. According to the latest epidemiological studies, the prevalence of major depressive disorder in Chinese children and adolescents has reached 1.9% to 2.1%, while the prevalence of bipolar disorder is about 0.8% to 0.9% [2]. Clinical manifestations of MDD are characterized by long-term depression, and loss of interest and pleasure, often accompanied by symptoms such as insomnia, loss of appetite, self-blame, and helplessness. By contrast, patients with BD experience manic episodes of elevated mood, excitement, and increased activity, as well as depressive episodes of low mood, fatigue, and helplessness [3]. These symptoms not only lead to impairment of their social functioning, affect the learning ability and social skills of children and adolescents, but also greatly increase the risk of suicide. Therefore, early identification and treatment are particularly important [1].

Currently, the diagnosis of these two mood disorders mainly relies on clinical interviews by physicians, collection of medical history data combined with auxiliary examinations, and finally diagnosis according to the Diagnostic and Statistical Manual of mental disorders (DSM) [4] and International Classification of Diseases (ICD) [5] diagnostic manuals. The diagnostic process is complicated and has some subjectivity by clinicians at all levels, easily leading to missed diagnoses and misdiagnosis of some patients [6]. By contrast, objective measures in terms of voice recognition have been proposed to contribute to the detection of depression. Voice recognition, or voiceprint, refers to the unique characteristics of an individual’s voice. They are created by capturing and analyzing various features of a person’s voice, such as pitch, tone, rhythm, and pronunciation. As a non-invasive and convenient experimental method, it holds the potential to assist in the diagnosis and monitoring of depressive disorders and bipolar disorders. While the voices of depressed patients often exhibit characteristics of dullness, reduced speech rate, monotony, and lifelessness [7], the voices of patients developing bipolar disorder symptoms may be characterized by increased speaking rate [8]. Hence, it has been proposed that this technology may facilitate the differentiation between MDD and BD.

Research using objective and scientific methodologies have also studied the voice prints of patients developing MDD and BP. However, earlier studies might be reductionist by relying on basic time domain features such as frequency, pitch, etc. [9] As features extracted from the time domain are highly affected by individual differences, their validity in predicting MDD and BD diagnoses may be limited. Nevertheless, research applying high-dimensional voice feature sets with a particular focus on the selection of more comprehensive features for emotion recognition is emerging. Advanced features, such as Mel-Frequency Cepstral Coefficients (MFCC), Mel frequency envelope spectra, short-time time domain features, fundamental frequency features, and emotion features, have been incorporated in recent research. Accordingly, more in-depth information about the voice features of the sound signals of patients with MDD and BD has been provided. To exemplify, Zhang et al. predicted the severity of suicide and depression in web recordings with an AUC of 0.821, from voice features including the spectral slope, MFCC, harmonic differences, pause length, pause-to-speech ratio, and pause variability [10]. Shinohara et al. also proposed an emotion index by voice characteristics to differentiate between 14 healthy individuals and 30 patients with major depression, with an AUC of 0.76 [11]. Shin et al. further studied the voice features of healthy subjects, patients with minor depressive episodes, and patients with major depressive episodes by extracting 21 speech features from semi-structured interview recordings. They identified 7 speech indicators that differed significantly across the three groups, with a sensitivity of 65.6%, specificity of 66.2%, and AUC of 65.9% by machine learning classification [12].

Similar studies have also been conducted on bipolar disorder. One experiment using voice feature such as pitch and variance extracted from naturalistic phone calls to discriminate between adults with MDD, BD and HC showed moderate accuracy [13]. In addition, employing a high-dimensional voice feature set used in emotion recognition, Higuchi et al. proposed a voice index capable of distinguishing between healthy individuals and patients with bipolar I or II disorder with an accuracy of about 67% [14]. Subsequently, the authors collected voice samples from 14 depressed patients, 8 bipolar disorder patients and 32 normal controls from Japan. The model was built by analyzing nine voice parameters including MFCC, fundamental frequency envelope and pulse code modulation, and the classification accuracy for the three groups was as high as 90.79% [15]. Taken together, analysis of speech parameters may distinguish bipolar disorder from depression. These studies show that vocal technology and artificial intelligence can help us better understand and identify mental illnesses.

Nevertheless, there are still some limitations in these studies. To start with, although many studies have classified disorders through machine learning, which often requires a certain sample size for accurate classification, the sample sizes involved are small. Second, current research has focused on adults, with less research on speech recognition for children and adolescents [16]. Children and adolescents with mood disorders demonstrate symptoms that differ from those seen in adults. For instance, children with MDD may not always exhibit the typical persistent low mood; instead, they may display irritability and tantrums. These atypical symptoms can contribute to increased speech rate and pitch in their voices, which may resemble the voice features of patients developing bipolar disorder [17]. However, to the best of our knowledge, no study has been conducted to compare the voice characteristics of the three groups of children and adolescents with MDD, BD, and healthy controls. Therefore, the generalization of existing diagnostic tools may be limited, and further study involving the youth population is needed. In our previous study, we explored the differences in voice features between children and adolescents with major depressive disorder and healthy controls, and established a multidimensional voiceprint feature assessment system with a sensitivity of 92.73% and a specificity of 90.91% for MDD through comprehensive feature screening [18]. The present study has been conducted based on previous studies, aiming to find vocal features that can be used to differentiate between three groups of children and adolescents with depressive disorder, bipolar disorder and healthy controls, and to achieve efficient identification of emotional disorders in children and adolescents. We first extracted multidimensional features from speech data, such as energy features, spectral features and rhythmic features, and calculated various statistical functions based on the extracted features to construct a more comprehensive feature set, which is more comprehensive and accurate than the previous extraction of speech features from the time–frequency domain. Then, we selected the features with large differences between classes through feature screening. Finally, the integrated classification method was used to achieve high classification accuracy.

## Methods

### Subject

50 inpatients with MDD and 50 with BD at Beijing Anding Hospital were recruited for the study. Subjects in the MDD group met the DSM-5 diagnostic criteria for MDD and those in the BD group met the DSM-5 criteria for BD. Both female and male participants were involved in the study, and all subjects were aged between 6 and 16. Subjects were only included if they were able to cooperate to complete the study, and had signed the informed consent prior to the study. The study adopted the exclusion criteria corresponding to our previous study [18]. Children and adolescents (a) with severe physical illness, (b) who demonstrated symptoms comorbid with other psychiatric disorders (e.g., schizophrenia, conduct disorder, personality disorder) and developmental disorders (e.g., autism spectrum disorder, intellectual disability), or (c) other conditions deemed inappropriate for inclusion in the group by the investigators were excluded. Additionally, 50 typically developing children and adolescents aged from 10 to 18 years with no other health conditions were recruited from schools and the community. Subjects were assessed for depressive symptoms and severity using the Hamilton Depression Rating Scale (HAMD) [19] and for manic symptoms and severity using the Young manic rating scale (YMRS) [20]. No subjects dropped out halfway through the trial. They were able to cooperate to meet all the experimental requirements. The project met the ethical standards set forth in the Declaration of Helsinki and its subsequent amendments and was approved by the Ethics Committee of the Beijing Institute of Technology (BIT-EC-H-2022120). All subjects and their families signed informed consent before the trial. During the trial, the subjects were able to cooperate with data collection and scale assessment requirements. The collected data were valid and reliable.

### Reading material

To protect subject privacy and confidentiality, and to ensure a standardized procedure, this experiment used a set consisting of seven pieces of reading materials to collect speech data. One short story (i.e., the North Wind and the Sun, see the Additional file 1), two positive emotional vocabulary lists, two negative emotional vocabulary lists and two neutral non-emotional vocabulary lists were included in the set (Table 1).

**Table 1:** Vocabulary lists used for recording

	Vocabulary List (Translated)	Purpose
1	Extraordinary, precious, comfortable, winning the prize, excellent, magnificent, expert, beautiful, winning, reunion	Emotional; positive
2	The middle, the central, the cause, in short, all levels, the backbone, the middle, among, all countries, all items	Non-emotional; positive
3	Drop the idea forever, drop the idea forever, misfortune, madness, howling, mourning, severe injury, depression, evil, disgust	Emotional; negative
4	Cheerfulness, perfection, ecstasy, infatuation, love, affection, congratulation, elite, super cool, sweet	Emotional; positive
5	Stockaded village, goods, mountain fastness, earliest, religion, shade, honor and disgrace, commodity, grape, talk about	Non-emotional; positive
6	Heartbreak, rape, resentment, humiliation, pain, misery, despair, sorrow, hatred, collapse	Emotional; negative

### Voice data

The experiment was conducted in a quiet indoor environment. The device we used to record was a MacBook Air 2020. After simple guidance from the staff, the subjects began to read the reading material aloud, while started recording. The whole process took about 3 min. During the recording, the subjects were required to avoid making other sounds as much as possible and keep a distance of about 30 cm between the mouth and the microphone.

### Voice feature

A basic pre-processing including power normalization and speech segmentation was adopted in this study. Power normalization was used to reduce the power differences between the recorded data and ensure the data comparability. Subsequently, each subject’s speech recording was segmented into seven parts, corresponding to the seven reading materials. The descriptors used in this study were INTERSPEECH 2016, which contains 6373 statistical features. These features were obtained by calculating various functions on the contours of low-level descriptors (LLD). This feature set covers a wide range of descriptors from speech processing, music information retrieval, and general sound analysis fields.

### Statistical methods

Demographic and clinical scale score data using Statistical Product and Service Solutions (SPSS) Version 18.0. The normality of the data distribution was assessed using the Shapiro–Wilk test (Shapiro and Wilk, 1965). One-way ANOVA and Tukey's post hoc test were used to compare parametric variables, and the Kruskal–Wallis test was used for non-parametric variables. In all experiments, p-values less than 0.05 were considered statistically significant. Feature selection in the feature set used the chi-square test to select the top 120 most effective features for differentiation of BD, MDD, and HC. In the selection of classification methods, we compared the machine learning algorithms including Decision Tree (DT), naïve Bayes Classifier (NB), Support Vector Machine (SVM), K-Nearest Neighbor (KNN) and Ensemble learning (EL), as well as the deep learning algorithms in terms of Convolutional Neural Network (CNN). We divided the human sample into training and testing sets in a 7:3 ratio, selected the optimal classification method in the 105 training sets, and finally calculated the diagnostic efficiency of the model using the 45 testing sets.

## Results

### Comparison of demographic and clinical symptom information

The demographic and clinical symptom data of all subjects are shown in Table 2. There were no significant differences in age and gender among the three groups (p > 0.05). There were significant differences in HAMD and YMRS scores among the three groups. The HAMD scores of the MDD and BD groups are similar. The MDD group took antidepressants and the BD group took mood stabilizers.

**Table 2:** Clinical and demographic characteristics

	MDD (n = 50)	BD (n = 50)	HC (n = 50)	*p*
Age (mean ± SD)	14.48 ± 1.752	14.24 ± 1.585	14.55 ± 2.661	0.322
Male (male/female)	18/32	13/37	21/29	0.236
HAMD (mean ± SD)	23.34 ± 4.706	21.04 ± 8.962	1.66 ± 0.760	< 0.001
YMRS (mean ± SD)	2.68 ± 0.884	9.38 ± 7.401	1.57 ± 0.827	< 0.001
Medication, n (%)				
Antidepressants	50 (100.00%)	13 (26.00%)	0	n/a
Antipsychotics	32 (64.00%)	20 (40.00%)	0	n/a
Mood stabilizer	18 (36.00%)	50 (100.00%)	0	n/a

*HAMD* Hamilton depression scale*;YMRS,* Young manic rating scale

### Feature selection

**Fig. 1:**
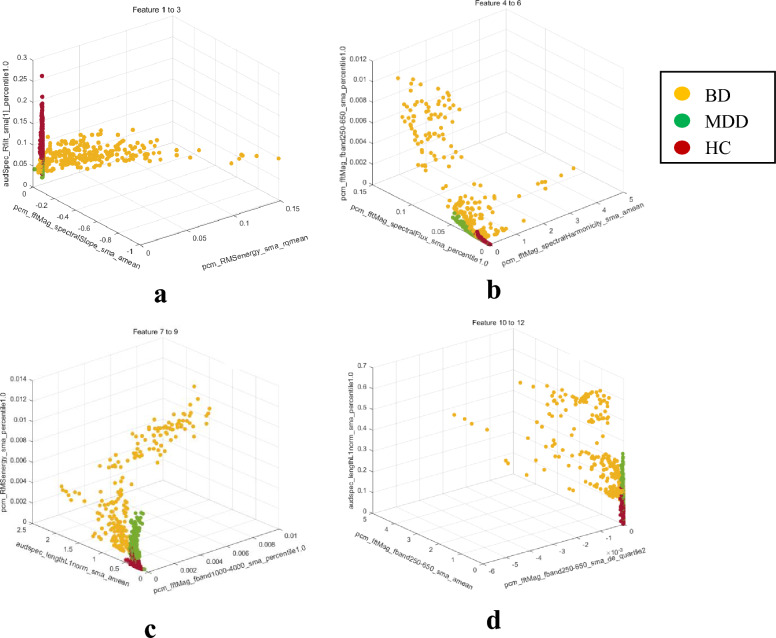
Clustering diagram among the three groups: *BD* bipolar disorder group; *MDD* major depressive disorder group; *HC* healthy control group. **a** different clusters among the three groups under features 1–3; **b** different clusters among the three groups under features 4–6; **c** different clusters among the three groups under features 7–9; **d** different clusters among the three groups under features 10–12

The chi-square test is a statistical method used to determine whether there is an association between classification variables. In the feature selection process, the correlation between them can be evaluated by calculating the chi-square statistic between features and the target variable. The feature selection process for the feature set involved in this study adopted the chi-square test, and we have ensured that the selected features maintain the highest possible correlation while minimizing redundancy and the risk of overfitting. Ultimately, our feature selection method led to the choice of 120 features for differentiating BD, MDD, and HC. The top 12 features with the highest scores are shown in Table 3. Through the difference visualization map drawn for the top 12 features, we observe that there are different cluster centers in the three groups in the case of multiple feature combinations, as shown in Fig. 1.

**Table 3:** Description of the top 12 vocal features

	Name of features	Meaning of features	*− log (P)*
1	pcm_RMSenergy_sma_rqmean	Represents the mean value of the root mean square (RMS) energy of a speech file	314.97
2	pcm_fftMag_spectralSlope_sma_amean	Represents the mean value of the slope of the power spectrum of the speech signal	303.08
3	audSpec_Rfilt_sma[1]_percentile1.0	Represents the energy magnitude of the signal in the 301–600 Hz frequency band in hundredths of a decimal place	303.01
4	pcm_fftMag_spectralHarmonicity_sma_amean	Represents the average value of the spectral harmonicity of the audio signal	297.07
5	pcm_fftMag_spectralFlux_sma_percentile1.0	Represents the 1.0 percentile of the spectral flux of the audio signal	294.77
6	pcm_fftMag_fband250-650_sma_percentile1.0	Represents the 1.0 percentile of the spectral amplitude of the audio signal in the frequency band 250 Hz to 650 Hz	294.17
7	pcm_fftMag_fband1000-4000_sma_percentile1.0	Represents the 1.0 percentile of the spectral amplitude of the audio signal in the frequency band 1000 Hz to 4000 Hz	289.21
8	audspec_lengthL1norm_sma_amean	Represents the average of the L1 parametres of the spectral length of the audio signal	288.56
9	pcm_RMSenergy_sma_percentile1.0	Represents the 1.0 percentile of the root-mean-square energy of the audio signal	288.3
10	pcm_fftMag_fband250-650_sma_de_quartile2	Represents the dichotomous difference of the spectral amplitude of the audio signal in the frequency band 250 Hz to 650 Hz	280.2
11	pcm_fftMag_fband250-650_sma_amean	Represents the average value of the spectral amplitude of the audio signal in the frequency band 250 Hz to 650 Hz	279.83
12	audspec_lengthL1norm_sma_percentile1.0	Represents the 1.0 percentile of the L1 parity of the spectral length of the audio signal	279.23

### Selection of training set classification model

A ternary classifier was used to verify the validity of the selected features, and the performances of DT, NB, SVM, KNN, EL and CNN were also evaluated. Speech accuracy, subject accuracy, AUC and time cost were used as evaluation metrics. Particularly, speech accuracy refers to the ratio of correct speech recordings from patients to all the speech recordings from all the subjects, and subject accuracy is defined as the ratio of correct diagnoses made on the different subjects. To explain, subjects would be considered to have been judged correctly if more than three out of seven of their speech recordings are judged correctly. To exclude anomalies, each algorithm was tested six times, and the average of the six times was calculated for all evaluation metrics, and the results are shown in Table 4, with SVM performing the best.

**Table 4:** The performance of various classifiers

Classifier	Speech accuracy (%)	Subject accuracy (%)	AUC	Time cost (s)
CNN	77.46	83.33	0.887	62.3988
DT	85.27	91.37	0.886	0.8865
NB	80.13	86.87	0.897	0.9364
SVM	91.98	94.75	0.991	0.8726
KNN	86.92	92.37	0.974	0.9324
EL	90.95	93.27	0.985	1.8739

*CNN* Convolutional Neural Networks*; DT* Decision Tree*; NB,* Naive Bayes Classifier*; SVM* Support Vector Machine*; KMN* K-Nearest Neighbor*; EL* Ensemble learning

### SVM classification results for the test set

**Fig. 2:**
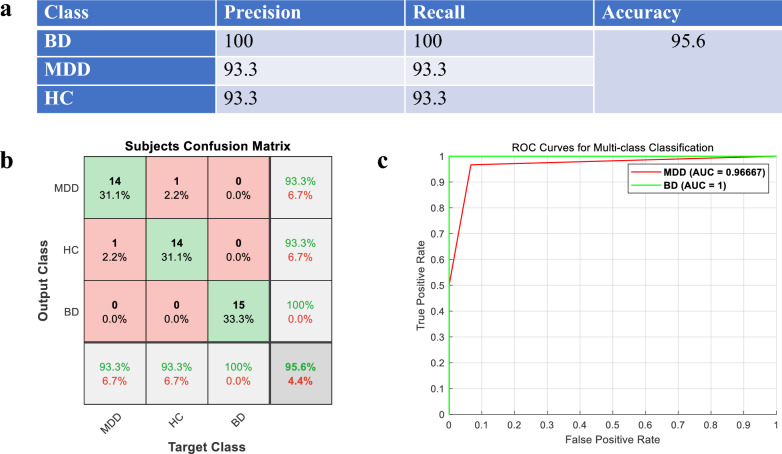
SVM classifier test set results. **a** Performance of classifiers; **b** confusion matrices of SVM classifier; **c** ROC curves of SVM classifier. *BD* bipolar disorder group; *MDD* major depressive disorder group; *HC* healthy control group

The classification results of the test set using SVM are shown in Fig. 2. Figure a shows the classification results of SVM, Figure b shows the confusion matrix, the total accuracy of SVM for the three groups was 95.6%, the sensitivity for MDD was 93.3%, the sensitivity for BD was 100% and the specificity was 93.3%. Fig. c was the ROC (receiver operating characteristic) curve of this classifier. The AUC for BD was 1 and the AUC for MDD was 0.967.

## Discussion

In view of the current difficulties in diagnosing mood disorders in children and adolescents and the lack of objective means, this study collected and analyzed the voice features of subjects under specific paradigms to effectively distinguish between children and adolescents with major depressive disorder (MDD) and bipolar disorder (BD) to assist in clinical diagnoses. In this study, 50 patients diagnosed with MDD according to the DSM-5 diagnostic criteria, 50 patients with bipolar disorder, and 50 healthy controls were recruited. Their clinical characteristics were assessed by the currently most commonly used HAMD scale and YRMS scale in clinics. Many patients with bipolar disorder had similar scores to patients with MDD on the HAMD scale. This finding again supports that the scales cannot explain the differences in the pathogenesis and physiological characteristics between bipolar disorder and depressive disorder [21], and there are high risk of subjectivity. As an objective physiological characteristic, the voice feature overcomes the shortcomings of scale assessment [22]. Based on the distinctiveness of MDD and BD symptomologies and previous findings on their voice feature differences, we hypothesized that despite patients with depressive disorder and bipolar disorder may perform similarly on the scale, there are differences in patients’ voice features [13]. Findings from this study confirmed our hypothesis.

The voice features obtained by voice extraction and voice screening are shown in Table 3. These 12 features can better distinguish the objective voice features of the three groups, mainly focusing on the root mean square energy, power spectral slope, 1% quantile energy in the low frequency band, and L1 norm of spectral harmonicity, spectral flux, spectral amplitude and spectral length. Through the combination of features, we can see that the three groups have significantly different cluster centers. As the root mean square energy reflects the overall intensity of the speech [23], these features would help to distinguish the three group. Patients with MDD and BD may experience abnormal changes in speech intensity due to physiological and emotional factors. Previous research has shown that patients with MDD speak with reduced intensity, reduced pitch range, and slower speaking rate [24]. Other research also identified speech energy features associated with MDD and BD, consistent with this study [25, 26]. Hence, these studies promote that root mean square energy is a sensitive indicator for distinguishing mood disorders.

The power spectral slope refers to the rate of change in the power of a signal as a function of frequency. Previous studies have reported that as the severity of depression increases, the spectral energy shifts from below 500 Hz to 500–1000 Hz [27, 28]. Upon relief of depression symptoms, the energy below 500 Hz increases, while the energy between 500–1000 Hz and 1000–1500 Hz decreases [29]. This shift of energy from low-frequency to high-frequency range in patients with depression may be the result of increased tension in the vocal cords and folds, which alters the resonance characteristics of the vocal cord filter and leads to a decrease in the spectral slope [30]. Moreover, excessive tension in the vocal cords and the lack of coordination among the muscles in the throat and articulation can interfere with the vibration of the vocal cords, causing irregular and erratic movements, which affects the stability and coherence of speech signals, leading to changes in the spectral harmonicity index. Spectral harmonicity refers to the harmonic relationship between different frequency components in speech signals and can be used to characterize the stability and coherence of speech signals [31]. Furthermore, the speech features selected in this study have been previously validated in many studies to be effective in distinguishing between various psychiatric disorders such as depression, bipolar disorder, and anxiety [32–34]. All of this evidence reinforces our belief that the combination of these features can be highly effective in the identification of mood disorders in children and adolescents, supporting the reliability of the final identification model built in this study.

During the training process of performing machine learning models, we found that Support Vector Machine (SVM) exhibited the best classification ability, which is related to the excellent performance of SVM on high-latitude data, high tolerance for noisy data, and the advantage that the training process can be highly optimized [35]. SVMs have also been reported in previous studies to perform better when classifying the severity of depression [36]. In contrast, algorithms such as Decision Tree (DT), K-Nearest Neighbor (KNN), and Ensemble Learning (EL) may experience overfitting or underfitting in certain situations, leading to a decrease in accuracy on test data. In addition, the computational complexity of the KNN and EL algorithms is also high, which may result in efficiency issues when processing large-scale data [37]. Therefore, we ultimately chose SVM to classify the test set, and the recognition model established by the SVM algorithm demonstrated excellent classification performance on the test set, with sensitivity, specificity, and diagnostic accuracy all above 90%, and the AUC of the ROC curve all above 0.95, indicating that the model has excellent diagnostic efficacy.

This study explored a voice feature dataset for emotional disorders in children and adolescents and established highly accurate recognition models for depression and bipolar disorder. Two main measures were taken to maintain the performances of replicability and robustness in clinical applications. Firstly, we adopted a feature selection method to reduce the number of features and ensure that the selected features have a satisfactory amount of information. We have also tried fewer features during the training and testing, whereas experiments have revealed that 120 features could achieve optimal classification performance. In other words, our findings support that reducing the number of features selected may lead to performance degradation. Secondly, regarding the replicability of the model, we also apply the proposed method to the public datasets (i.e., MODMA and DAIC-WOZ). The accuracy of the proposed method in distinguishing different types of data in these two datasets were higher than 80%.

However, there are still some limitations. To start with, the sample size is relatively small, and the number of datasets used for testing is limited. Further experiments may expand the sample size to further optimize the model. Moreover, this study did not classify diseases in detail. In further research, we will further expand the sample size, classify the participants according to emotional state, and further divide them into multiple groups such as MDD-mild, moderate, and severe, and BP-1, 2 types, etc., not only for the auxiliary diagnosis of diseases but also for more accurate judgment of the participants' current emotional state. The assessment of patients' emotional state by objective voice features has great advantages over traditional scales, and its objectivity and simplicity are very easy to promote in the clinic. In addition, the model in this study does not require high voice quality and is appropriate for remote medical systems, and for early identification of patient diseases and health monitoring. Patients can record their voices in a home environment, and the system can determine the patient's emotional state, which can then be remotely evaluated by a clinical doctor. This will greatly improve medical efficiency and is therefore worth promoting in the medical and health fields.

## Conclusion

We explore the characteristics of voice features in children and adolescents and used machine learning to achieve effective differentiation of depression and bipolar disorder in children and adolescents. The established model has sensitivity and specificity of more than 90%, proving that voice features can be used as an effective objective physiological indicator for the diagnosis of emotional disorders. The findings of the study facilitate the use of voice features in clinical practice as an auxiliary diagnostic tool for emotional disorder. However, we also recommend research on stakeholders’ acceptance of the devices using this technology to be conducted. Qualitative research on the attitude and user experience of among psychiatrists, clinicians, user and the general public may be particularly meaningful in evaluating the usefulness of the tools.

### Supplementary Information

**Additional file 1.** Reading material.

## Data Availability

The data that support the findings of this study are available on request from the corresponding author. The data are not publicly available due to privacy or ethical restrictions.
