# Supplementary material for: Differentiation between depression and bipolar disorder in child and adolescents by voice features
Source: Child Adolesc Psychiatry Ment Health. 2024 Jan 29;18:19. doi: 10.1186/s13034-024-00708-0 (PMC10826007; doi:10.1186/s13034-024-00708-0)
Supplement: Supplementary file 1 — Additional file 1. Reading material. [file 13034_2024_708_MOESM1_ESM.docx]

***Reading paradigm-Translation version***

1. Once, the north wind and the sun argued about who was more capable. They happened to see a man walking by, who was wearing a cloak. They said whoever can let the man remove his coat, even if he is more capable. So the north wind began to blow hard. However, the harder he blew, the more the man wrapped himself in his cloak. Finally, the north wind had no choice but to give up. Then, when the sun came out, the man felt very hot and immediately took off his coat. So the north wind had to admit defeat.
2. extraordinary, precious, comfortable, winning the prize, excellent, magnificent, expert, beautiful, winning, reunion
3. the middle, the central, the cause, in short, all levels, the backbone, the middle, among, all countries, all items
4. drop the idea forever, drop the idea forever, misfortune, madness, howling, mourning, severe injury, depression, evil, disgust
5. cheerfulness, perfection, ecstasy, infatuation, love, affection, congratulation, elite, super cool, sweet
6. stockaded village, goods, mountain fastness, earliest, religion, shade, honor and disgrace, commodity, Grape, talk about
7. heartbreak, rape, resentment, humiliation, pain, misery, despair, sorrow, hatred, collapse

***Reading paradigm-Original version***

1.有一次，北风和太阳正在争论谁比较有本事。他们正好看到有个人走过，那个人穿着一件斗篷。他们就说了，谁可以让那个人脱掉那件斗篷，就算谁比较有本事。于是，北风开始拼命地吹。怎知，他吹得越厉害，那个人就越是用斗篷包紧自己。最后，北风没办法，就放弃了。接着，太阳出来晒了一下，那个人感觉很热，就立刻把斗篷脱掉了。于是，北风只好认输。

2.不凡、宝贝、自在、中奖、极好、恢弘、高手、美好、优胜、团圆

3.正中、中央、因为、总之、各级、中坚、中部、中间、各国、各项

4.死心、丧气、不幸、抓狂、嚎哭、报丧、重创、抑郁、邪恶、厌恶

5.欢畅、绝佳、狂喜、迷恋、酷爱、钟情、贺喜、精英、超爽、甜蜜

6.寨子、货物、山寨、最早、宗教、树阴、荣辱、商品、提子、谈起

7.心碎、强奸、怨恨、作贱、苦痛、凄惨、绝望、忧愁、痛恨、崩溃
